# Patient safety awareness among Undergraduate Medical Students in Pakistani Medical School

**DOI:** 10.12669/pjms.342.14563

**Published:** 2018

**Authors:** Rizwana Kamran, Attia Bari, Rehan Ahmed Khan, Mohamed Al-Eraky

**Affiliations:** 1 Dr. Rizwana Kamran. MBBS, MHPE. Medical Educationist, University of Lahore, Lahore, Pakistan.; 2 Dr. Attia Bari. MBBS, DCH, MCPS, FCPS, MHPE. Associate Professor, Department of Pediatric Medicine, The Children’s Hospital Lahore, Lahore, Pakistan.; 3 Dr. Rehan Ahmed Khan. MBBS, FCPS, FRCS, JM-HPE, MSc HPE. (Professor of Surgery), Riphah International University, Rawalpindi, Pakistan.; 4 Dr. Mohamed Al-Eraky. MBBCh, MSc, MMEd, PhD. Zagazig University, Egypt, Assistant Professor in Medical Education, University of Dammam, Saudi Arabia

**Keywords:** Patient safety, Medical errors, Medical students, Attitude

## Abstract

**Objective:**

To measure the level of awareness of patient safety among undergraduate medical students in Pakistani Medical School and to find the difference with respect to gender and prior experience with medical error.

**Methods:**

This cross-sectional study was conducted at the University of Lahore (UOL), Pakistan from January to March 2017, and comprised final year medical students. Data was collected using a questionnaire ‘APSQ- III’ on 7 point Likert scale. Eight questions were reverse coded. Survey was anonymous. SPSS package 20 was used for statistical analysis.

**Results:**

Questionnaire was filled by 122 students, with 81% response rate. The best score 6.17 was given for the ‘team functioning’, followed by 6.04 for ‘long working hours as a cause of medical error’. The domains regarding involvement of patient, confidence to report medical errors and role of training and learning on patient safety scored high in the agreed range of >5. Reverse coded questions about ‘professional incompetence as an error cause’ and ‘disclosure of errors’ showed negative perception. No significant differences of perceptions were found with respect to gender and prior experience with medical error (p= >0.05).

**Conclusion:**

Undergraduate medical students at UOL had a positive attitude towards patient safety. However, there were misconceptions about causes of medical errors and error disclosure among students and patient safety education needs to be incorporated in medical curriculum of Pakistan.

## INTRODUCTION

Patient Safety (PS) has emerged as an essential healthcare discipline in medical curricula to reduce the rate and effect of adverse events.^1^–^3^ The iconic report “To Err is Human”, reported shocking statistics on deaths among Americans because of medical errors compared to deaths with automobile accidents and claimed a total of almost 100,000 deaths per year in the United States due to medical errors.^4^

In Pakistan, many patients die and face other serious health problems because of the poorly structured healthcare system and lack of focus on PS protocols.^5^ Reforms in the policies of Pakistan Medical and Dental Council are pertinent on the basis of current changes globally. The changes in medical curriculum are also imperative to meet the new standards of current medical education.^5^ The level of error in health care and lack of awareness of its significance draws the attention of medical educators to address the issue of PS. Several researchers have emphasized the importance of incorporating PS education in graduate medical education.^6^,^7^ The World Health Organization (WHO) also recognized PS importance and integration into medical education and published a PS curriculum guide for medical students.^8^

Many questionnaires have been developed to investigate medical students’ perceptions and attitudes towards PS, namely: Madigosky et al questionnaire^9^, APSQ.^10^ These questionnaires provide the baseline data and information regarding PS to advise instructional designers to plan develop and implement appropriate educational programs for the curriculum.^2^,^3^,^11^–^15^

In Pakistani medical curricula PS is not given due importance to make students aware of PS issues. We planned this study to measure the level of awareness towards PS among Undergraduate Medical Students (UGMS) in Pakistani medical school and to find the difference with respect to gender and prior experience with medical error.

## METHODS

This cross-sectional study was carried out from January to March 2017 at the University College of Medicine and Dentistry, the constituent college of the UOL Pakistan. Census sampling was used for the study, 150 final year medical students from UOL were included, and 122 students returned the questionnaire with the response rate of 81%. Any foreign student because of their different previous experience were excluded from the study. The study was conducted after taking the permission from Institutional Review Board of the UOL and the developer of validated questionnaire APSQ-III through e-mail. Questionnaires were circulated to students and were told about the confidentiality and anonymity of the data. Seven-point Likert scale was used for the responses on items. With 7 being “Strongly Agree”, 4 “Unsure”, 1 being “Strongly Disagree”. For items (11, 13-18, 25), the scoring was reversed ranging from 1 (strongly agreed) to 7 (strongly disagreed). The scoring of individual response was classified as a “positive” response if the response was “strongly agree, agree or somewhat agree) in positively worded questions and (strongly disagree, disagree and somewhat disagree) in reverse coded questions.

Data analysis was done by using SPSS version 20. Independent sample t test was used to find the difference of attitudes in two groups with gender and prior experience. The statistical significance was set at a value ≤ 0.05.

## RESULTS

A total of 122 students out of a total of 150 registered in final year UGMS at UOL, participated in the study with the response rate of 81%. Distribution of the students by gender and previous experience with medical error is given in Table-I. Eighteen items showed positive attitudes, one item showed neutral attitude and seven items showed negative attitudes towards the PS factors and are shown in Table-II.

**Table-I:** Demographic characteristics of participants in the study n= 122.

Variables		Number of Students	Percentage of Students
Gender	Male	40	32.8%
	Female	82	67.2%
Prior Experience with Medical Error	Yes	40	32.8%
	No	82	67.2%

**Table-II:** Attitude of Pakistani under Graduate Medical Students’ towards PS.

Key Factors	Item No.	Item Using a 7-point Likert scale (7-strongly agree to 1-strongly disagree)	Mean Score/ SD
1. PS training received	1	My training is preparing me to understand the causes of medical errors	5.99 ± 0.95
	2	I have a good understanding of PS issues as a result of my undergraduate medical training	5.47 ± 1.16
	3	My training is preparing me to prevent medical errors	5.71 ± 1.10
	4	I would feel comfortable reporting any errors I had made, no matter how serious the outcome had been for the patient	5.08 ± 1.54
2. Error reporting confidence	5	I would feel comfortable reporting any errors other people had made, no matter how serious the outcome had been for the patient	4.96 ± 1.57
	6	I am confident I can talk openly to my supervisor about an error I had made even if it resulted in potential or actual harm to my patient	5.25 ± 1.24
	7	Shorter shifts for doctors will reduce medical errors	6.02 ± 1.24
3. Working hours as an error cause	8	By not taking regular breaks during shifts, doctors are at an increased risk of making errors	6.24 ± 1.18
	9	The number of hours’ doctors work increases the likelihood of making medical errors	5.86 ± 1.38
	10	Even the most experienced and competent doctors make errors	5.69 ± 1.36
4. Error inevitability	11	A true professional does not make mistakes or errors (R)	4.03 ± 2.00
	12	Human error is inevitable	5.25 ± 1.58
	13	Most medical errors result from careless nurses (R)	2.75 ± 1.33
5. Professional incompetence as an error cause	14	If people paid more attention at work, medical errors would be avoided (R)	1.83 ± 0.96
	15	Most medical errors result from careless doctors (R)	2.73 ± 1.47
	16	Medical errors are a sign of incompetence (R)	2.86 ± 1.46
	17	It is not necessary to report errors which do not result in adverse outcomes for the patient (R)	3.86 ± 1.82
6. Disclosure responsibility	18	Doctors have a responsibility to disclose errors to patients only if the errors result in patient harm (R)	3.37 ± 1.33
	19	All medical errors should be reported	5.93 ± 1.17
	20	Better multidisciplinary teamwork will reduce medical errors	6.24 ± 0.73
7. Team functioning	21	Teaching students teamwork skills will reduce medical errors	6.11 ± 0.93
	22	Patients have an important role in preventing medical errors	5.26 ± 1.37
8. Patient involvement in reducing error	23	Encouraging patients to be more involved in their care can help to reduce the risk of medical errors occurring	5.99 ± 0.92
	24	Teaching students about PS should be an important priority in medical students training	6.34 ± 0.79
9. Importance of PS in the curriculum	25	PS issues cannot be taught, they can only be learned through clinical experience, which is gained when one is qualified (R)	2.75 ± 1.65
	26	Learning about PS issues before I qualify will enable me to become a more effective doctor	6.21 ± 0.90
		

Positive attitude = score >4, Neutral attitude (N) = score 4, Negative attitude = score <4.

The mean scores of nine areas of PS shown in Table-III. Results showed that participants strongly supported the integration of PS curricula in undergraduate medical education. Majority of students acknowledged the role of multidisciplinary teamwork in reducing the occurrence of errors. Approximately, two out of every three students reported understanding of error inevitability and long working hours as causes of error. Students low score for disclosure or reporting responsibility also highlighted the area for improvement.

**Table-III:** Nine domains measuring attitudes towards PS.

S. No	Key factors	Item number	Score of key factor domain	Mean / SD
1	PS training received	1–3	17.17	5.72 ± 2.5
2	Error reporting confidence	4–6	15.29	5.09 ± 3.7
3	Working hours as an error cause	7–9	18.12	6.04 ± 3.0
4	Error inevitability	10–12	14.97	4.99 ± 2.9
5	Professional incompetence as an error cause	13–16	10.37	2.59 ± 3.5
6	Disclosure responsibility	17–19	13.16	3.00 ± 3.3
7	Team functioning	20-21	12.35	6.17 ± 1.4
8	Patient involvement in reducing error	22 -23	11.25	5.62 ± 1.9
9	Importance of PS in the curriculum	24–26	15.3	5.10 ± 2.2

SD: Standard deviation.

There was no significant difference seen in awareness of students with respect to their gender, prior experience with medical error and perception of PS in most of the items (P > 0.05).

## DISCUSSION

Unsafe patient care and medical errors resulting in adverse events in hospitalized patients are a global concern. PS awareness among healthcare providers is essential and central element in providing quality health care. The current study showed positive attitudes of UGMS towards PS, by gaining 70% score in the questionnaire. This is in accordance with a study from USA, which also indicated similar findings.^12^ Most students believed that PS education is taught only in clinical settings. Majority of students in the present study reported attitudes supportive to statements regarding the PS training they received. A study in Singapore discloses a finding consistent with present study, students indicated positive attitudes to this domain.^14^ As PS is not a formal topic in the undergraduate curriculum, this positive attitude could be the reason of job training in their clinical settings.

Disclosure responsibility showed second lowest score among the domains. Although majority agreed with reporting all medical errors but almost one third of the participants were less positive with reporting medical errors as shown by low scores in reverse coded question 3.86 and 3.37. This showed the knowledge gap on what is considered as reportable about medical errors. This could be the reason of faulty error reporting system or lack of proper education about patient safety issues in Pakistani curricula. Health professionals may avoid facing the problems of litigation due to reporting of errors. The development of error reporting system in health care institutions and protecting doctors from litigation is a vital factor to promote the culture of PS and is yet to be developed.^16^

Due to round the clock functioning of hospitals’ the doctors are often sleep deprived, fatigued and exhausted because of their long job duration.^17^ Majority of students reported that tired health professionals may be more prone to errors. This may be indicative of the students putting stress on the individual approach which in line with the findings of published studies involving students from all over the world.^1^,^2^,^10^

Team collaboration is essential as PS is at risk when health care professionals are not communicating properly. In our study, the domain “Team Functioning” scored the highest points showed that students recognized the vital role of teaching teamwork for error reduction. Other studies also reported similar results for this domain and scored highest in KSA and USA.^2^,^12^ Students involved in the present study acknowledged the role of patients in reducing errors. Similar findings were reported in various studies.^10^,^13^

A large number of students recognized the significance of PS education, and strongly agreed its inclusion in the medical curriculum. This is similar to the findings from other studies in Pakistan and other countries.^2^,^3^,^13^ There is a basic misunderstanding about the teaching of PS reflected in one item by agreeing that PS issues cannot be taught, they can only be learned through clinical experience.^10^ In Pakistan, PS is taught informally only in clinical years which could be the reason of this perception of medical students.

The domain ‘Professional Incompetence as an Error Cause’ in reverse coded question showed lowest score because majority of participants in the current study believed that individual failures are the main causing factors in the occurrence of most errors. This finding is similar to the findings from Pakistan and Hong Kong.^1^,^3^ This domain is a clear indication that the inclusions of these topics in future programs or modules are needed for UGMS.

### Implications

Teaching error reporting as an integral element of communication skills and the total working hours of doctors should not be more than eighty hours per week.

### Strengths and Limitations

A validated and a reliable questionnaire (APSQ III) was used. The limitation of this study is that it is done in only one private institution of Pakistan. There is a need to replicate the current study in other private and public medical schools for the generalization of results.

## CONCLUSION

Under graduate medical students showed positive attitude towards patient safety analyzed through this questionnaire supporting the inclusion of PS education, team work approach and patient involvement. There is a big knowledge gap found among students about the causes of medical errors and responsibility of error disclosure. PS education is inadequate and needs to be incorporated in medical curriculum of Pakistan.

### Authors’ Contribution

**RK:** Data collection, statistical analysis & manuscript writing.

**AB:** Conceived, designed & final editing of manuscript.

**RAK:** Revised the work critically.

**MAE:** Revised it critically for important intellectual content.
